# Effect of Plasma Treatment on Coating Adhesion and Tensile Strength in Uncoated and Coated Rubber Under Aging

**DOI:** 10.3390/ma18020427

**Published:** 2025-01-17

**Authors:** Miguel Angel Martínez, Juana Abenojar, Daniel García-Pozuelo

**Affiliations:** 1 Materials Science and Engineering Department, Universidad Carlos III de Madrid, 28911 Leganés, Spain; 2 Mechanical Engineering Department, Universidad Carlos III de Madrid, 28911 Leganés, Spain; dgramos@ing.uc3m.es

**Keywords:** rubber, coating, aging, UV radiation, atmosphere pressure plasma

## Abstract

The degradation of rubber materials under environmental and mechanical stress presents a significant challenge, particularly due to UV (ultraviolet light) exposure, which severely impacts the material’s physical properties. This study aims to enhance the UV stability and longevity of rubber by evaluating the performance of modified polyurethane and silicone coatings as protective stabilizers. Natural rubber—styrene–butadiene rubber (NR-SBR), known for its exceptional mechanical properties, was selected as the base material. To ensure strong adhesion, cold atmospheric plasma treatment was applied, increasing the surface energy by 250%, primarily through an enhancement of the polar component. After treatment, supplier-recommended coatings were applied and tested for adhesion using the pull-out method. Aging tests under UV exposure, water immersion, and high temperatures were conducted to assess durability, with tensile tests used to monitor changes over time. Coatings exhibiting cracking after UV exposure were excluded from further analysis. A silicone coating demonstrating superior moisture resistance and durability under extreme conditions was identified as a promising candidate for future UV stabilization applications. These findings provide a foundation for developing advanced coatings to significantly extend the service life of rubber materials in demanding environments.

## 1. Introduction

Elastomeric materials have many applications in high-consumption industries, such as tires, conveyor belts, and shock absorbers, and in emerging ones such as dielectric elastomer actuators [1], and even in the biomedical industry [2].

There are numerous elastomer polymer compositions that can be utilized in the tire industry, but the most commonly employed are natural rubber and synthetic rubber. Depending on the specific type, these materials offer high slip resistance and, after processing, the desired elasticity. Tires typically incorporate a blend of natural rubber (NR) and synthetic rubber. Among synthetic rubbers, common types include styrene–butadiene rubber (SBR), butadiene rubber (BR), isobutylene–isoprene rubber (IIR), and isoprene rubber (IR). The composition mix can vary across industries [3]. For passenger vehicle tires, SBR is generally the primary component, with BR as a secondary material. However, for truck, bus, or aircraft tire casings, NR is predominantly used due to the higher strength-to-deformation ratio required in these applications [4].

Elastomers are susceptible to aging, a process that gradually degrades material properties. This degradation occurs as rubber polymers undergo chemical interactions with the surrounding environment. Over time, these interactions can lead to the formation of additional crosslinks or the breaking of molecular backbones and existing crosslinks, leading to a decline in performance [5,6,7]. To illustrate this, some examples of degradation include rubber aging as an irreversible phenomenon, with the rate of deterioration influenced by various factors such as temperature, ultraviolet (UV) light exposure, and humidity, among others [5]. For instance, nitrile rubber (NBR) tires are prone to cracking due to temperature effects. As temperature increases, the hardness of the rubber increases while stress/strain capacity decreases. At 70 °C, heat accelerates oxidation, confirming temperature as a key aging factor [6].

Furthermore, during the initial stages of aging, moderate crosslinking may slightly improve tensile strength. However, prolonged aging causes over-crosslinking and uneven distribution, weakening mechanical properties. Over time, the compression set increases as chain fractures inhibit elastic recovery. These effects bring about residual deformation under compressive and thermal oxidative conditions, particularly at elevated temperature of 125 °C, 135 °C, and 150 °C [7], compromising the material’s ability to recover its original shape and adversely affecting its long-term mechanical performance.

Similarly, in carbon black-filled styrene–isoprene–butadiene rubber (SIBR-CB), thermal–oxidative aging at 90–130 °C revealed a wide-ranging aging temperature coefficient due to polybutadiene and polyisoprene chains being prone to crosslinking and chain scission [8]. Crosslinking is the dominant process, which helps extend the material’s shelf life. Additionally, photodegradation depends strongly on temperature, while humidity has a minimal influence [9].

In styrene–butadiene rubber (SBR), thermo-oxidative aging (50–100 °C for 7–60 days) exhibited similar trends. Aging reduced mechanical properties and molar mass between crosslinks, while increasing crosslinking density and hardness. Chemical changes, detected by ATR-FTIR, were predominantly caused by thermo-oxidation, especially at higher temperatures and longer exposure. A threshold molar mass indicated complete degradation, while time–temperature equivalence modeling effectively predicted stress and strain at break [10].

UV radiation impacts ethylene–propylene–diene monomer (EPDM) rubber by triggering surface oxidation, which forms oxygenated species and increases microcracks over time. Initially, chain scission reduces crosslink density, increasing free volumes and gas permeability. After 14 days, crosslinking dominates, reversing these trends and improving properties like tensile strength, glass transition temperature (T_g_), and storage modulus, indicating an increase in the material’s stiffness [11].

Thermal oxidation and UV aging in natural rubber (NR) revealed significant changes in surface morphology, molecular structure, and mechanical properties. Thermal aging accelerates UV-induced surface cracks and deepens discoloration, while the combined effects degrade tensile strength and elongation at break by damaging molecular bonds and crosslinking points. This synergistic mechanism produces aldehydes, ketones, and esters, highlighting the interaction of heat and UV radiation in NR aging [12].

In summary, numerous studies on various rubber compositions have consistently found that aging, driven by factors such as temperature, UV radiation, and oxidation, leads to changes in mechanical properties, molecular structure, and surface morphology, with effects ranging from initial strengthening due to crosslinking to eventual degradation from chain scission and over-crosslinking. Despite these insights, extending the lifecycle of rubber materials remains a challenge, particularly in the case of tires. Tires, one of the largest contributors to global waste generation, are often discarded in both controlled and uncontrolled landfills. As products made from non-renewable fossil fuels, tires are not easily recyclable. Disposal typically involves burning, a process that releases substantial amounts of CO_2_ and other greenhouse gases, exacerbating environmental harm [13]. Every day, researchers work to find solutions for reducing this waste [14]. Tires are made from a complex mixture of materials, including elastomers, fillers, and various chemical additives [13,15]. These additives play essential roles in production, such as vulcanization agents—commonly sulfur or organic compounds—and accelerators or activators like zinc oxide, magnesium oxide, amines, and benzothiazoles. These substances optimize the vulcanization process by reducing the required amount of vulcanizing agent or delaying the reaction for better control. Vulcanization, which forms covalent crosslinks in the rubber’s molecular structure, enhances chemical resistance, thermal stability, and mechanical strength. However, the durability of vulcanized elastomers presents a major challenge for the disposal and recycling of elastomeric products. Tires may also include carbon black and silica. Adding more silica improves fatigue resistance and rolling resistance and reduces heat buildup, but reduces tensile strength, Young’s modulus, and wet grip [16], contributing to increasing the amount of waste and greater environmental pollution as the tire wears down.

Various approaches are being explored to address the challenges of aging and recycling waste in elastomers. In terms of recycling and environmental impact, bio-based elastomer composites offer an alternative to fossil fuel–derived materials, providing a renewable alternative for tire production [17]. The grinding process and separation of the tire material yield raw materials, such as rubber, steel, and fiber particles, which can be reused in various industrial applications [18,19]. In this context, Zanchet et al. [20] studied SBR compounds with industrial rubber residues, ground at ambient conditions and devulcanized via microwaves (SBR-r), as reinforcing fillers, demonstrating good performance after vulcanization.

Emerging trends also encompass cutting-edge innovations, including reversible dynamic chemical crosslinking techniques. These approaches allow elastomers to self-repair when damaged, significantly prolonging their lifespan and supporting a circular economy [21,22,23,24].

Another promising approach involves the ECOTIRE project, which is pioneering a more sustainable and innovative tire design. This project proposes a smart tire with a removable tread that can be replaced with a new one, effectively extending the life of the tire casing and reducing overall waste [25]. The design retains rubber casing while replacing the tread material with one that has less impact on the environment and human health, such as silicone. To further enhance durability and minimize environmental impact, a specialized coating is proposed for the casing, providing added protection and extending its usability. The application of this coating to tire casing represents the future application and novelty of this study.

The focus of this research is on the use of polyurethane and silicone-based coatings to protect NR-SBR rubber from environmental factors such as temperature, humidity, and UV radiation. The application of protective coatings to rubber is a promising way to extend the material’s lifespan and reduce waste. Additionally, the concept of intelligent tires often involves the instrumentation of the casing, making any improvement in the casing’s life crucial to the industrialization and actual use of smart tires in the future.

## 2. Materials and Methods

This research is structured around several key stages that guide the investigation of the effectiveness of polyurethane- and silicone-based coatings in protecting elastomers from environmental factors. The methods employed in this study were designed to comprehensively evaluate the performance of these coatings, particularly in terms of their durability and resistance to UV radiation, temperature, and humidity. The following stages outline the approach taken:

1. Market Analysis of Coating Materials: The first step involves identifying commercially available coatings, with a focus on evaluating their viscosity to ensure ease of application and performance under UV radiation exposure.
2. Adhesion Characterization: Given the challenge posed by the low wettability of rubber, this stage examines various adhesion treatments. Pull-out tests with different coatings are used to evaluate the strength and reliability of these treatments.
3. Characterization of Treatment: Further characterization is performed using techniques such as contact angle measurements, surface energy analysis, and X-ray photoelectron spectroscopy (XPS) to gain insight into the surface properties and treatment effects.
4. UV Resistance Evaluation: This phase compares the behavior of coated and uncoated rubber samples when exposed to UV radiation over extended periods, providing valuable information on the coatings’ protective capabilities.
5. Aging Analysis Under Controlled Conditions: To simulate real-world conditions, the study investigates rubber degradation at various temperatures and humidity levels. Specific temperature and humidity combinations are selected to assess how these factors contribute to the aging process.
6. Performance Testing of Selected Coating: Once the most promising coatings are identified, they are applied to rubber samples and subjected to aging tests under the selected temperature and humidity parameters. This includes testing both individual and combined environmental factors.

To assess the impact of aging, various evaluation techniques were used, including hardness tests, tensile tests, and colorimetry.

### 2.1. Materials

Experimentally, an NR-SBR-type material was used for this purpose, supplied by Eguía Manufacturas de Goma, S.L. (Madrid, Spain). This material exhibits excellent mechanical properties and is suitable for elastic and cushioning applications. A sheet with a thickness of 10 mm, a width of 1000 mm, and a length of 10 m (reference 0910.001) was cut into samples measuring 50 mm × 50 mm, as well as into dog bone-shaped samples with a length of 115 mm.

Regarding the coatings, polyurethanes and silicones were selected based on the supplier’s recommendations. For confidentiality, these coatings are referred to as PU_1, PU_2, and so on for polyurethanes and Si_1, Si_2, etc., for silicones. Only the specific coating selected is identified in detail within the paper. Initial tests were conducted using seven different coatings, with their main characteristics listed in Table 1.

Then, a liquid silicone for elastic coatings, based on MS technology (modified silane), curing at room temperature upon contact with air humidity, was used for waterproofing and roof repair. This silicone demonstrated better protection compared to the previous coatings listed in Table 1. It exhibits excellent resistance to UV radiation and weathering. The silicone was supplied by Quilosa Professional (Quilosa—Selena Iberia S.L.U., Madrid, Spain) under the trade name Aqua Protect. This silicone corresponds to Si_4 in Table 1.

### 2.2. Surface Treatment

A cold atmospheric pressure plasma torch (AAPT) from Treat GmbH (Steinhagen, Germany) was used to treat the rubber samples. The system operated at a frequency of 17 kHz with a high-voltage discharge of 20 kV and featured a rotating torch equipped with a nozzle (1900 rpm) for plasma ejection. The setup included an electronically speed-controlled stage to hold the samples during treatment. Cold air plasma was generated at a working pressure of 200 kPa within the rotating nozzle through an imbalance discharge and was expelled through a circular orifice onto the sample surface. Treatment parameters were optimized by varying the stage speed and the distance between the plasma torch nozzle and the samples, with water contact angle measurements used to determine optimal conditions. The ideal parameters were identified as a stage speed of 10.5 mm/min and a nozzle-to-sample distance of 10 mm. After treatment, the samples were stored under dust-free conditions until adhesion tests were performed.

### 2.3. Characterization of Surface Treatment

#### 2.3.1. Contact Angle and Surface Energy

Contact angle measurements were performed both before and after plasma treatment to assess the wettability of the rubber. An OCA 15 plus device from DataPhysics (Neurtek Instruments, Eibar, Spain) was used, following the UNE-EN 828 standard [26]. Prior to measurement, the samples were placed in an isothermal chamber at (24 ± 2) °C, pre-saturated with vapor of the corresponding liquid for at least 10 min. Liquid drops were deposited on the surface, and the contact angle was recorded within 1 min of application.

The surface tension of the test liquids—bidistilled deionized water, diiodomethane, and glycerol—was determined using the pendant drop method [27]. For each sample, at least five drops were analyzed and averaged using the sessile drop technique. The solid surface free energy components were calculated using the Owens–Wendt–Rable–Kaelble (OWRK) model [28], a widely used method for analyzing the contact angle with several liquids. This approach enables the determination of the dispersive component (attributable to London forces) and the polar component (arising from dipole–dipole interactions and hydrogen bonding) by fitting the linear form of the equation to the experimental data. The OWRK method is particularly suitable for this study, as it provides a detailed understanding of the role of polar and dispersive interactions in wettability and adhesion, which are critical factors in evaluating coating performance on rubber surfaces.

#### 2.3.2. Pull-Off Test

Adhesion pull-off tests were conducted on rubber using stud or dolly aluminum joints, with the coating serving as the adhesive, according to the UNE-EN ISO 4624 standard [29]. The adhesion performance of the coating was compared between untreated rubber and rubber treated with APPT. The coating itself serves as an adhesive for bonding with the dolly. The dollies had a diameter of 20 mm, and the load values (N) provided by the machine were converted to tensile stress values (MPa). The treatment for the dollies involved cleaning, sanding, and the application of Sikaprimer^®^–207, specified for aluminum. For each selected coating (Table 1), five samples were tested, both with and without treatment.

#### 2.3.3. X-Ray Photoelectron Spectroscopy (XPS)

The surface chemical composition of the rubber was investigated using X-ray photoelectron spectroscopy (XPS), both before and after plasma treatment. An ellipsoid scan probe with a major diameter of 400 μm, calibrated with the carbon peak (C-C/C-H, 284.6 eV), was employed for the analysis. XPS measurements were performed on a Kratos XSAM 800 spectrometer (Kratos Analytical Ltd., Shimadzu Group, Manchester, UK) in fixed analyzer transmission mode, with excitation provided by Mg Kα (1253.6 eV). Survey spectra were acquired in the kinetic energy range of 0–1300 eV, with 0.5 eV increments. High-resolution photoelectron lines of key elements, including O1s, N1s, C1s, and S2p, were recorded with 0.1 eV steps. It should be noted that XPS primarily analyzes the surface composition, as it probes a depth of approximately 10 nm.

### 2.4. Aging of Untreated Rubber and Coating Rubber

#### 2.4.1. UV Radiation

The accelerated aging test for color loss due to sunlight exposure was conducted using a Solarbox 3000E chamber equipped with a 2500 W Xenon lamp, supplied by Neutrek (Guipúzcoa, Spain), following the standard UNE-EN ISO 4892-3 [30]. The black standard temperature (BST) was set to 65 °C, with a radiation intensity of 550 W/m^2^. The samples were exposed in the chamber for a maximum of 1128 h (47 days), simulating over two years of outdoor aging.

#### 2.4.2. Humidity and Temperature

The rubber samples, both coated and uncoated, were subjected to aging at 40 °C, 60 °C, and 90 °C. Test temperatures of 40 °C, 60 °C, and 90 °C were selected to represent a range of conditions relevant to real-world tire usage. While 40 °C simulates minimal aging effects, 60 °C reflects typical tire temperatures during normal driving, and 90 °C represents elevated conditions such as those experienced during sudden stops or low-pressure driving. These values also align with findings from previous studies [7,8,11].

Durability tests were conducted using a climatic chamber, which consisted of a sealed polypropylene container equipped with an airtight closure system. This chamber was placed inside an oven (Digitronic TFT—JP Selecta S.A., Barcelona, Spain). For tests evaluating the combined effects of humidity and temperature, water was introduced into the chamber, ensuring that the samples remained above the liquid without direct contact, maintaining the chamber humidity between 97% and 100% RH.

Environmental conditions were monitored throughout the experiment using a thermo-hygrometer (MSR 145—MSR Electronics GmbH—Seuzach, Switzerland), allowing precise recording of relative humidity (RH) and the set temperature. To assess the adhesive bond’s degradation, tests were carried out on samples exposed to two or three different aging durations and then compared against non-aged materials. The exposure time varied depending on the specific property being measured and the material’s degradation level.

### 2.5. Characterization of the Aging Process in Uncoated and Coated Rubber Samples

The measured properties included color, weight loss, hardness, and tensile strength, which were used to calculate maximum strength, elongation, and Young’s modulus.

#### 2.5.1. Study of Color

Given the importance of aesthetics, the initial evaluation focused on assessing the color of the sintered materials and its variation from the base material, which in this case was coated rubber. Color systems take into account three essential components: the light source, the observer, and the object. The CIELab system, endorsed by the International Commission on Illumination, UNE-EN-ISO/CIE 11664-2 standard [31] and widely recognized worldwide, was used for this analysis, UNE-EN-ISO/CIE 11664-4 standard [32].

In the CIELab system, colors are represented using three coordinates: L*, a*, and b*. Additionally, chroma (C*) indicates the saturation or intensity of the color (Equation (1)), while the hue angle (*h*) corresponds to the specific chromatic tone (Equation (2)). The asterisk (*) denotes that these values adhere to the CIELab standard. To measure these parameters, a ColorEyé XTH device provided by Neurtek Instruments (Eibar, Guipúzcoa, Spain) was utilized.

(1) $$C^{*}={\left(a^{2}+b^{2}\right)}^{1/2}$$

(2) h=arctanab

#### 2.5.2. Weight Loss and Hardness Test

The Shore A hardness (HSA) of rubber samples was evaluated before and after aging using a Shore durometer (Bareiss, Oberdishingen, Germany) in compliance with the UNE-EN ISO 863 standard [33]. Measurements were taken on samples with a minimum thickness of 6 ± 0.2 mm. The scale reading was recorded after 15 ± 0.1 s, with six measurements per specimen, spaced 6 ± 0.2 mm apart and 9 ± 0.2 mm from the edge. Each test involved three specimens per material.

#### 2.5.3. Tensile Test

The tensile test was conducted using a universal testing machine from Microtest S.A. (Madrid, Spain) equipped with a 5 kN load cell and operating at a speed of 10 mm/min. The dog-bone-shaped sample dimensions were 115 ± 5 mm in total length and 30 ± 4 mm in width at the heads, with a calibrated section measuring 20 ± 0.5 mm in width and 50 ± 0.25 mm in length, in accordance with ASTM D638 standard [34].

#### 2.5.4. Infrared Spectroscopy

The technique most frequently used is Fourier transform IR spectroscopy (FTIR). The FTIR spectra of the rubber samples before and after UV radiation aging were recorded using an infrared spectrometer machine (Bruker Optik GmbH, Ettlingen, Germany) equipped with an attenuated total reflection (ATR) technique to analyze the surface chemical modifications. The produced spectra were collected with a Bruker Tensor 27 spectrometer at the resolution of 4 cm^−1^, 32 scans, and an incident radiation angle of 45°. Three spectra were captured for each rubber to ensure homogenous results.

## 3. Results

### 3.1. Optimization and Characterization of Surface Treatment

**Figure 1:**
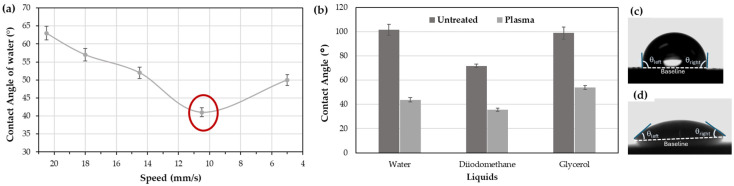
(**a**) Optimization of the APPT treatment rate with water droplets (the red circle corresponds to the best condition.); (**b**) contact angles before and after APPT treatment with water, diiodomethane, and glycerol, (**c**) water droplet on untreated rubber; and (**d**) water droplet on APPT-treated rubber. The baseline and the left and right angle are specified in (**c**,**d**).

The APPT surface treatment was optimized at 10.5 mm/s by adjusting the distance to the torch and the plasma speed. Figure 1a illustrates how the contact angle decreases as the speed decreases, reaching a minimum at 10.5 mm/s, after which it begins to increase again. A similar approach was taken for the distance: the speed was fixed at 10.5 mm/s, and the distance was reduced until the optimal value of 10 mm was identified. The optimization was performed using contact angle measurements with water.

Figure 1b shows the contact angles obtained with various liquids with different surface tensions. The most dispersive liquid, diiodomethane, exhibits higher wettability, consistent with the surface’s low wettability properties. Figure 1c displays the contact angle before plasma treatment, while Figure 1d shows the angle after treatment. A significant reduction in the contact angle is observed, indicating an increase in surface wettability, which allows the liquid to spread more effectively.

**Figure 2:**
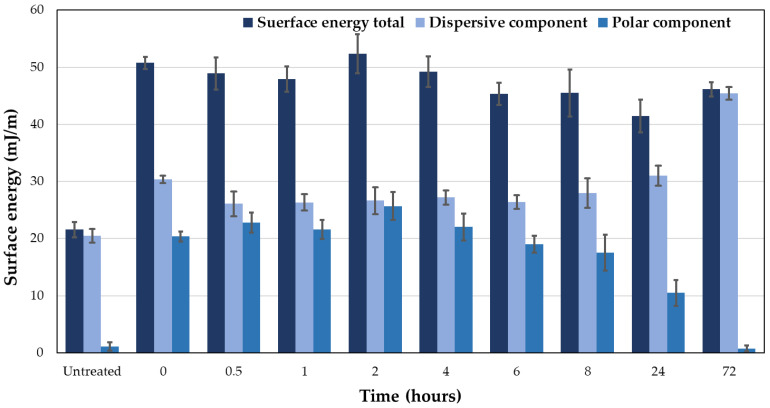
Evolution of surface energy and its polar and dispersive components of rubber over time.

Using the contact angles of the three liquids, the equipment software calculates the surface energy of the material (Figure 2). The results show that the surface energy significantly increases after APPT treatment, with a 58% rise in total surface energy for the rubber. More notably, the polar component of the surface energy increases by 95%, which is critical, as this component enhances the polar character of the surface, improving its ability to attract polar adhesives or coatings.

The surface energy remains high for up to two hours after treatment and even shows a slight increase of 2%. However, after two hours, the surface energy gradually declines, reaching its lowest point at 72 h. At this stage, the surface retains high dispersive energy but lacks polar energy. Therefore, it is recommended to apply the coating within the first four hours following APPT treatment for optimal results.

**Figure 3:**
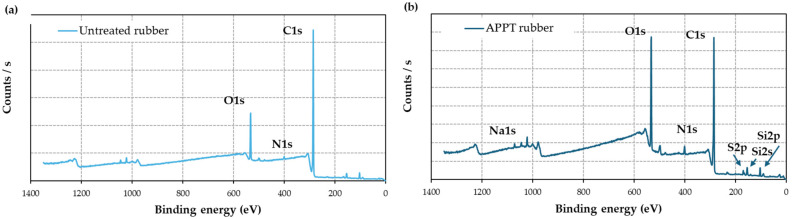
XPS wide scans for (**a**) untreated rubber and (**b**) APPT-treated rubber.

XPS provides information about the elements present and the types of bonds formed between these elements. Figure 3a shows that the primary elements are C, O, N, Si, and S. After treatment, the peak heights increase, making O, N, S, and Si more prominent (Figure 3b). As a result, the percentage of these elements changes, and the ratio to carbon decreases (Table 2). Both silicon and sulfur are additives in rubber.

The decrease in carbon is primarily due to the removal of surface contaminants and the elimination of carbon black, the main additive in rubber. This also increases the visibility of silicon and sulfur.

**Figure 4:**
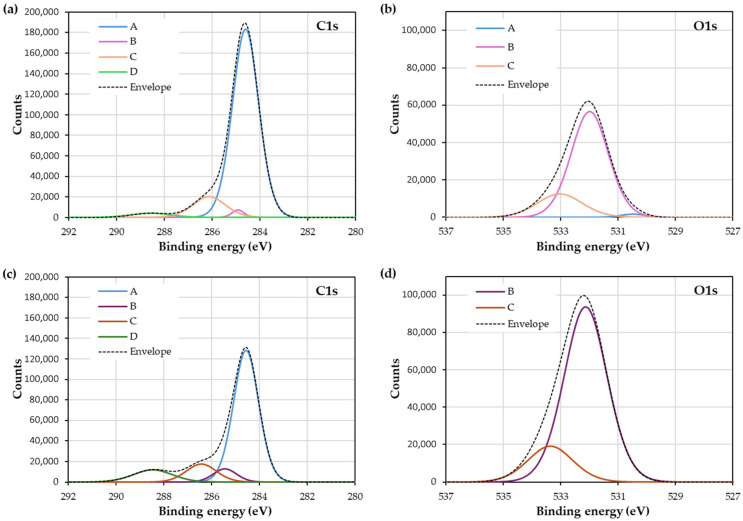
Deconvolution of XPS peaks for untreated rubber: (**a**) C1s peak and (**b**) O1s peak; for treated rubber: (**c**) C1s peak and (**d**) O1s peak.

The deconvolution of these major peaks reveals the types of bonds formed between the elements. Figure 4 shows the deconvoluted peaks for untreated rubber (C1s in Figure 4a and O1s in Figure 4b) compared with those for treated rubber (C1s in Figure 4c and O1s in Figure 4d).

The deconvolution of the C1s peak for untreated rubber (Figure 4a) shows four peaks at 284.59 eV, 284.91 eV, 286.12 eV, and 288.57 eV. The first two peaks (A and B), corresponding to C-C and C-H bonds, account for 48.16% and 43.42%, respectively. These are likely associated with the rubber polymer chain or carbon black, especially in the case of the second peak. The third peak (C), at 7.18%, may correspond to C-OH bonds, but could also indicate C-S bonds, as their binding energies are very close. Finally, the fourth peak (D), at 1.24%, could be attributed to -C=O, C-N, or C-S bonds [35,36].

The deconvolution of the O1s peak (Figure 4b) reveals three peaks associated with silicon—SiO_2_ (7.30%) peak A, Si-O-Si (62.75%) peak B, and SiOH (29.95%) peck C—with bending energy at 530.47 eV, 531.98 eV, and 533.02 eV [37].

For rubber treated with APPT, the C1s deconvolution (Figure 4c) shows four peaks at 284.56 eV (A), 285.45 eV (B), 286.43 eV (C), and 288.47 eV (D). These correspond to C-C and C-H bonds (62.47%), -C=O bonds (22.52%), C-OH and C-N bonds (10%), and a combination of -C=O, C-N, and C-S bonds (6%) [35,36,38].

The O1s peak for treated rubber (Figure 4d) can also be deconvoluted into three peaks. The first peak at 530.57 eV, corresponding to SiO_2_, is not visible in the graph. The second peak at 532.14 eV peak A, corresponds to C-OH bonds, and the third peak at 533.37 eV, peak C, is associated with C=O bonds [35,36].

In summary, untreated rubber contains C-C/C-H, C-S, and C-N bonds, which correlate with the presence of sulfur and nitrogen. For nitrogen, C-N and R-C≡N bonds are observed at 398.42 eV and 399.61 eV, respectively [39,40]. The third peak corresponds to the quaternary ammonium ion (NH_4_^+^) at 401.59 eV. In the case of treated rubber, C-N and C=N bonds are identified at 398.78 eV and 399.99 eV [39,40], respectively. The third peak also corresponds to the NH_4_^+^ ion [41]. Both doublets are observed in untreated and treated rubber.

**Figure 5:**
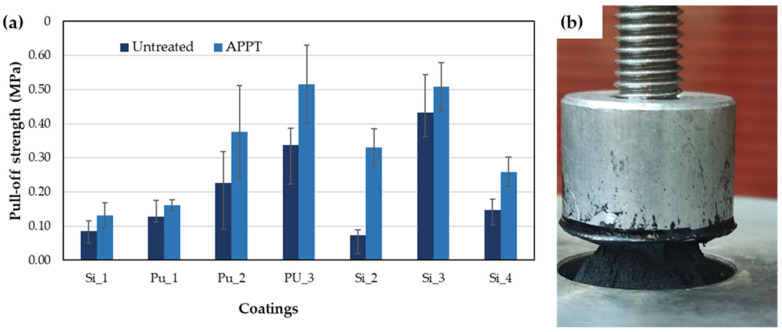
(**a**) Pull-off strength of untreated and treated rubber coated with silicones and polyurethanes listed in Table 1; (**b**) pull-off test for Si_4 coating on treated rubber.

**Figure 6:**
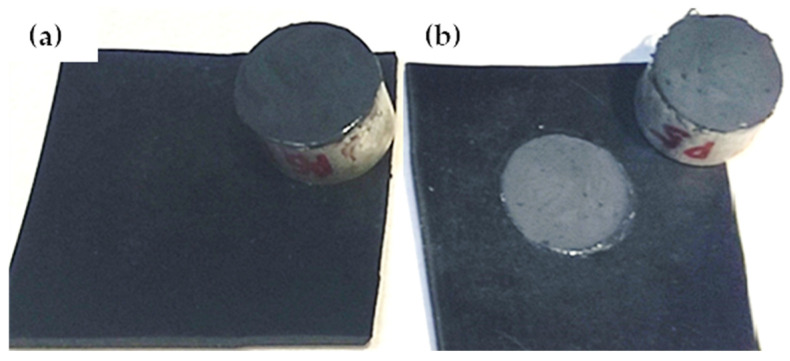
(**a**) Adhesive failure mode for untreated rubber coated with Si_3 and (**b**) cohesive failure mode for rubber treated with APPT.

The pull-off tests performed on the coated rubber samples indicate improved resistance following APPT treatment of the rubber, regardless of the coating type studied (Figure 5). Prior to treatment, all failures were adhesive (Figure 6a), except for PU_1, which was uncured. After the APPT treatment, the failures were predominantly cohesive (Figure 6b), except for Si_1, which exhibited mixed failure, and the uncured PU_1.

### 3.2. Coating Selection

Considering the coatings initially selected (Table 1), this section focuses on choosing a single coating. The first selection criterion was UV radiation behavior, and the second was ease of application. For this part of the study, rubber samples were prepared for tensile tests, and five samples were coated with each type of coating, comparing them to uncoated rubber.

**Figure 7:**
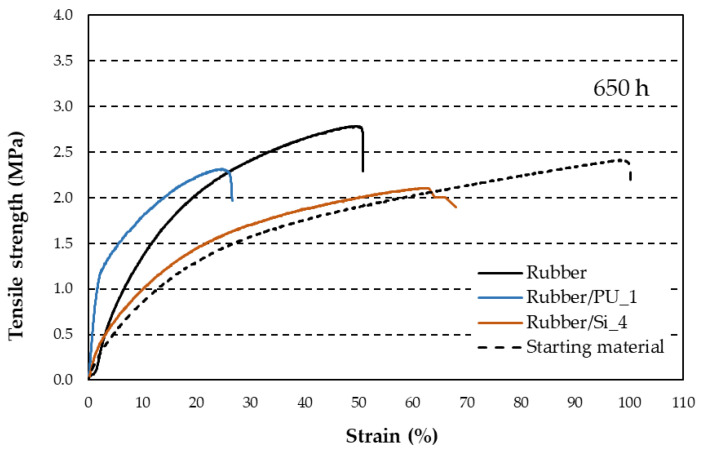
Tensile strength after 650 h of aging in a UV chamber. The starting material is untreated rubber without any coating, never aged. The sample represented is the one that comes closest to the average value of the five tested.

Figure 7 presents the behavior of one of the silicones (Si_4) and a polyurethane (PU_1) after 650 h of aging in a UV chamber. Their performance is compared to the initial uncoated rubber, tested before exposure to the UV chamber. The behavior of the silicones was very similar overall; Si_4 represented an average curve, as did PU_1.

Uncoated rubber hardens significantly, increasing its strength by 12.5%, while losing 50% of its strain. When comparing silicone coating to polyurethane, silicone loses 12.5% of its strength but maintains a large strain, although it is 30% lower than the starting material. Polyurethane loses less strength (6.25%) but becomes much stiffer, losing around 70% of its strain, and even performs worse than the uncoated material as it cracks and appears to be broken. Because of this, silicone was preferred over polyurethane, and due to its ease of application and resistance, Si_4 was selected. As previously mentioned in Section 2.1, it corresponds to the Aqua Protect (AP) silicone from Quilosa—Selena Iberia S.L.U.

### 3.3. Aging of Rubber and Coated Rubber Exposed to External Agents

**Figure 8:**
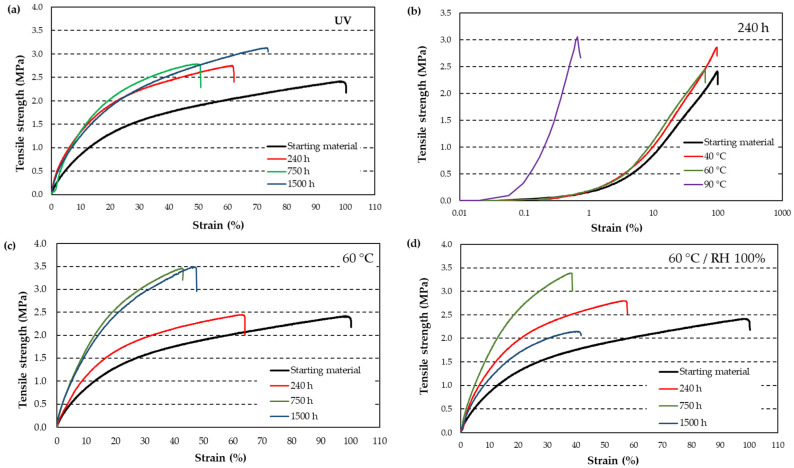
Tensile strength of rubber: (**a**) variation with UV radiation over time; (**b**) variation with temperature after 240 h; (**c**) variation over time at 60 °C; (**d**) variation over time at 60 °C and 100% RH. The sample represented is the one that comes closest to the average value of the five tested.

The effects of UV radiation, temperature, and the combination of temperature and humidity were studied through tensile tests. The curves shown represent the average of five tests per condition, aiming to provide representative values. Figure 8a illustrates the variation in rubber properties under UV radiation exposure. An increase in tensile strength of 12.5%, 15.4%, and 27.8% was observed for 240 h, 750 h, and 1500 h, respectively, in the UV chamber. Simultaneously, a loss of elasticity of 40% and 50% was noted for 240 h and 750 h, respectively, while at 1500 h, the loss of elasticity reduced to 28%.

Figure 8b depicts the effect of temperature on aging, with the *x*-axis plotted on a logarithmic scale. A temperature of 90 °C was excluded from further analysis due to the rapid degradation of material properties. Among the tested conditions, 60 °C was selected as a more realistic temperature to which the wheels might be exposed, making it the basis for subsequent aging tests.

For aging at 60 °C (Figure 8c), the effects are similar to those observed under UV exposure: an increase in tensile strength accompanied by a loss of elasticity. The tensile strength increased by 1%, 41%, and 44% after 240 h, 750 h, and 1500 h, respectively. Correspondingly, elasticity decreased by 36%, 56%, and 53% for the same durations.

The combined effect of 60 °C and 100% relative humidity (Figure 8d) revealed an increase in tensile strength at 240 h and 750 h, with gains of 15% and 40%, respectively. However, at 1500 h, a 13% loss in tensile strength was observed. Elasticity consistently decreased across all durations, with reductions of 42%, 61%, and 58% for 240 h, 750 h, and 1500 h, respectively.

**Figure 9:**
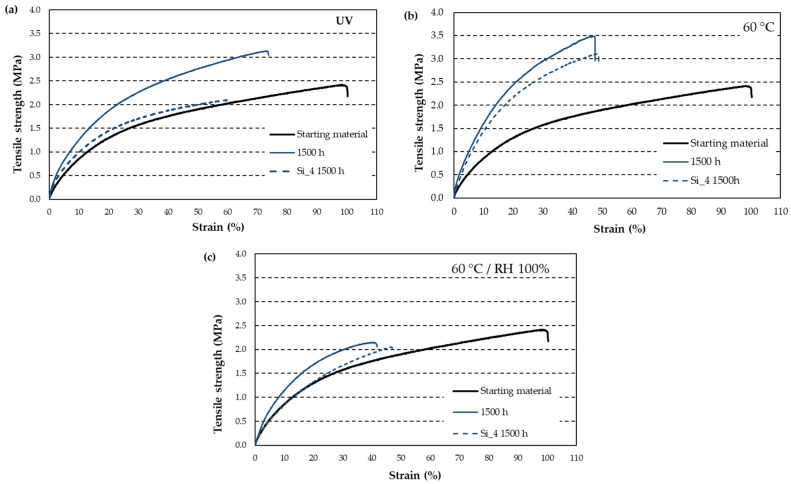
Comparison of the tensile strength of rubber with Si_4-coated rubber: (**a**) variation with UV radiation over time; (**b**) variation over time at 60 °C; (**c**) variation over time at 60 °C and 100% RH. The sample represented is the one that comes closest to the average value of the five tested.

A test duration of 1500 h was selected to evaluate the effect of silicone coating. Figure 9a shows that the coating prevents the increase in tensile strength observed in uncoated rubber and even results in a 13% decrease in strength, along with a 39% loss of elasticity. In contrast, uncoated rubber at the same time interval exhibits a 28% increase in strength, with a 27% reduction in elasticity.

At 60 °C, the Si_4 coating results in a 28% increase in strength, compared to a 41% increase for uncoated rubber after 1500 h. However, the loss of elasticity remains nearly unchanged, with values of 49% for uncoated rubber and 47% for coated rubber (Figure 9b).

The Si_4 coating provides better protection for the rubber against the combined effects of temperature and humidity (Figure 9c). It increases the rubber’s stiffness compared to uncoated rubber, while slightly enhancing its elasticity. However, at the breaking point, the coated rubber exhibits a 17% decrease in tensile strength and a 53% loss in elasticity, in contrast to a 13% decrease in strength and a 58% loss in elasticity for uncoated rubber.

As the temperature increases, rubber loses elasticity (Table 3). It also becomes slightly stiffer, although, according to the measurement error, it practically does not change, and all the values fall within the same range. As shown in Figure 8b, 90 °C is too aggressive a temperature, while 40 °C results in a small variation. Moreover, considering an average temperature at which asphalt may be found, a temperature of 60 °C was chosen for the rubber aging process.

The aging of rubber under UV radiation (Table 4) results in an increase in tensile strength over time by 12%, 16%, and 20% at 240 h, 750 h, and 1500 h, respectively. However, deformation decreases by 35%, 52%, and 30% for the same time intervals. The material becomes stiffer and loses elasticity. Interestingly, at 1500 h, the rubber seems to recover part of its elasticity.

The effect of temperature is less uniform (Table 4). At 240 h, tensile strength increases by 16%. At 750 h, it decreases by 4%, only to increase again by 24% at 1500 h. Elasticity, on the other hand, decreases by 36% and 60% for the first two time intervals, but recovers by 5% at 1500 h. Overall, the trend indicates that the material becomes stiffer and loses elasticity over time.

The combined effect of heat and humidity follows a similar pattern (Table 4). Tensile strength increases by 16% and 36% at 240 h and 750 h, respectively, but decreases by 12% at 1500 h. Deformation decreases by 42% and 61% for the first two time intervals, but shows a slight recovery of 2% at 1500 h.

Under ultraviolet radiation, silicone coatings help maintain tensile strength (Table 5), although elasticity decreases by approximately 45%. In contrast, uncoated rubber, as previously observed (Table 4), regains some elasticity, though it experiences a 30% loss overall.

Regarding temperature (Table 5), after 1500 h of aging, uncoated rubber resembles coated rubber at 1000 h. Its tensile strength increases by 24%, with elasticity slightly below 50%. However, at 1500 h, the coated rubber exhibits the same tensile strength as the control rubber, while its elasticity remains around 50%.

The combined effect of temperature and humidity (Table 5) also shows that uncoated rubber at 1500 h is comparable to coated rubber at 1000 h. It experiences a tensile strength loss of approximately 12% and an elasticity loss of 59%. By 1500 h, tensile strength increases by 12%, while elasticity recovers slightly, resulting in a total loss of 51%.

Regarding color, Table 6 shows that uncoated rubber tends to shift towards the positive region of the b* axis, moving towards yellow, and shifts to the positive region of the a* axis, moving towards red. However, slight changes in lightness (L*) are observed, along with variations in chroma or color saturation (C*), with minor changes in the hue angle or actual chromaticity (*h*).

With the PU coating, a* and b* become more positive at 240 h of exposure, while at 1500 h, they shift back to the negative region. Slight changes are observed in L*, with significant variations in C* and *h*. In contrast, rubber coated with silicone remains in the same region of the axes, in the negative part of a* and b*, with a slight increase in L*. Chroma (C*) remains unchanged, as does the hue angle (*h*).

Finally, the hardness (Table 7) increased slightly in the uncoated rubber after 1500 h in the UV chamber, while the polyurethane-coated rubber showed a 22% increase. In contrast, silicone-coated rubber exhibited the opposite behavior, with a 67% decrease after 1500 h.

## 4. Discussion

### 4.1. Optimization and Characterization of Surface Treatment

The decrease in the water contact angle observed in Figure 1a to 10.5 mm/s, followed by an increase at lower speeds, is attributed to polymer migrations caused by the presence of waxes. When surface treatment with plasma is too intense, these waxes migrate to the surface, increasing the contact angle due to the dispersive nature.

APPT treatment is well known for introducing polar groups into polymer surface, a behavior particularly noticeable in apolar materials such as polyolefins [42]. It is also widely used for treating natural fibers [43,44]. Natural rubber and synthetic SBR lack polar groups in their chains, but both contain reactive double bonds. The APPT treatment induces chain cleavages in the surface chains and the formation of functional groups like hydroxyl and aldehydes. The treatment uses atmospheric air with nitrogen and oxygen to generate plasma, resulting in polar groups that enhance the surface’s polar character. Additionally, plasma flux effectively cleans the surface, removing contaminants and carbon black. This trend is supported by the observed decrease in the quantity of C1s (Figure 3, Table 2 and Figure 4a,c). The removal of surface carbon black further exposes other elements present, such as sulfur and silicon.

XPS analysis reveals predominant bonds in untreated rubber, such as C-C/C-H, C-S, and C-N, though the latter overlaps with C-O bonds. After APPT treatment, oxidation occurs, introducing bonds such as C-OH and C=O, confirmed through oxygen analysis (Figure 3, Table 2 and Figure 4b,d).

Sulfur is linked to carbon, forming bonds such as C-S-C, C-S, C-N, and R-C≡N. Additionally, nitrogen is present in the form of NH_4_^+^, derived from atmospheric nitrogen. Although this overlaps with N-O bonds [45], the latter are ruled out, as no corresponding peaks appear in the oxygen spectrum.

In both treated and untreated rubber, oxygen bonds are associated exclusively with silicon. Silica, widely used in tire manufacturing, improves elongation at break and reduces weight. Silica integration is facilitated by silane coupling agents, which enhance dispersion and mechanical properties [46,47]. XPS analysis of silanes identifies Si-O-Si (bridge oxygen) bonds and SiO^−^ (terminal oxygen) structures, supporting their role in coupling [37].

The APPT treatment also increases the pull-off strength of coated rubber (Figure 5a), while additionally causing a change in the failure mode from adhesive to cohesive (Figure 6).

### 4.2. Coating Selection

Polyurethanes typically exhibit poor performance against UV radiation. For this reason, primers are commonly applied to the windscreens of cars and trains to shiel the polyurethane surface from sunlight. However, modern polyurethanes modified with silanes (MS) show improved UV resistance. Even so, silicones generally exhibit better resistance to radiation, a property that is further enhanced when modified with silanes. For this reason, silicone (Si_4) was selected.

Despite advances in polyurethane technology, recent studies have shown that polyurethanes tend to undergo a color change when exposed to sunlight, shifting to yellow due to the oxidation of the methylene group in diphenylmethane diisocyanate. This is triggered by the breakdown of aromatic rings due to UV exposure [48,49,50].

Figure 7 shows that polyurethane-coated rubber exhibited less deformation and stiffer behavior compared to silicone-coated rubber. Although silicone-coated rubber showed less deformation, its curve mirrored that of uncoated rubber.

As seen in Table 6, polyurethane-coated rubber experienced greater color variations compared to silicone-coated rubber. Over time, the polyurethane coating showed a decrease in color intensity at 240 h and an increase at 1500 h, which resulted in a noticeable shift in the hue angle (*h*).

The color shift is due to changes in the a* and b* axes, which represent the hue of the color. The third axis, L*, measures lightness, while the C* (chroma) vector lies in the a* and b* plane, forming an angle (*h*) with the a* axis. As h increases, C* shifts closer to b*, resulting in a yellowish hue [32].

According to Table 6, uncoated rubber shifted towards the plane between red and yellow, with slight increases in h, but polyurethane-coated rubber showed more prominent yellowing due to an increase in h. In contrast, the silicone-coated rubber exhibited no significant color changes.

Table 7 also considers hardness. Polyurethane-coated rubber increased in hardness after 1500 h, indicating a loss of elasticity. In contrast, silicone-coated rubber became more elastic, which suggests that changes in rubber contraction or expansion will not significantly affect the silicone coating. However, such changes can lead to cracks or detachment in polyurethane coatings.

Due to these factors, polyurethane coatings were discarded, and the study proceeded with the silicone coating Si_4, which was easier to apply and demonstrated mechanical behavior equal to or slightly better than the other silicones tested.

### 4.3. Comparative Discussion on Rubber Aging: Infrared and Tensile Properties

The variability in the tensile test results, depending on the aging parameters, must be analyzed by considering the effects on polymer chains. Infrared spectroscopy is utilized here as a supporting tool to identify functional groups in these chains through their vibrations at specific wavelengths, providing insight into the chemical changes that justify the observed mechanical behavior. The light beam’s power can penetrate up to 10 μm into the sample, allowing the observation of the rubber’s functional groups, even in coated samples, although these functional groups may be masked by those of the coating.

#### 4.3.1. Functional Group Identification with FTIR

**Figure 10:**
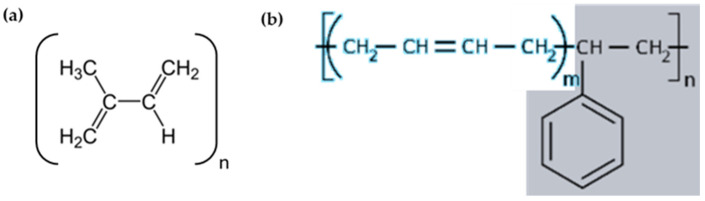
Starting monomers of the rubber used in this work: (**a**) NR (trans-isoprene (2-methyl-1,3-butadiene)) and (**b**) SBR (styrene marked in gray and butadiene written in blue).

According to Figure 10, the FTIR shows the vibrations corresponding to

- C-H of the methyl groups (-CH and -CH_2_) and vinyl groups (-CH=CH_2_-),
- (-C-C-) vibrations of the aliphatic chain and of the aromatic rings,
- (-C=C-) vibrations of the aliphatic chain and of the aromatic rings, and
- C-S or C-S-C groups and Si-O or Si-O-Si groups (detected in smaller proportions in the rubber, according to XPS analysis).

In summary, these vibrations provide a molecular-level understanding of the changes occurring in rubber due to aging. For example,

- In the region from 500 to approximately 1000 cm^−1^, deformation, bending, rocking, or wagging vibrations associated with C-H bonds are prominent, together with vibrations of groups (-CH=CH-) by C-H.
- Between 1030 and 1075 cm^−1^, small peaks correspond to the symmetrical stretching of the C-S and C-S-C groups or primary alcohols (R-CH_2_-CH_2_-OH).
- Stretching vibrations of methylene groups (-CH_2_), both symmetric and antisymmetric, appear as doublets in the range between 2800 and 3000 cm^−1^ [51].
- Hydroxyl groups from ambient humidity are in the range between 3000 and 3500 cm^−1^ [52]
- Between 1400 and 1500 cm^−1^, a relatively broad peak relative to the aromatic ring (-C=C-) and possible carbon black are observed [53].
- The region between 1600 and 1700 cm^−1^ includes vibrations of carbonyl groups (-C=O) and conjugated double bonds.

#### 4.3.2. Observations and Correlation with Mechanical Properties

**Figure 11:**
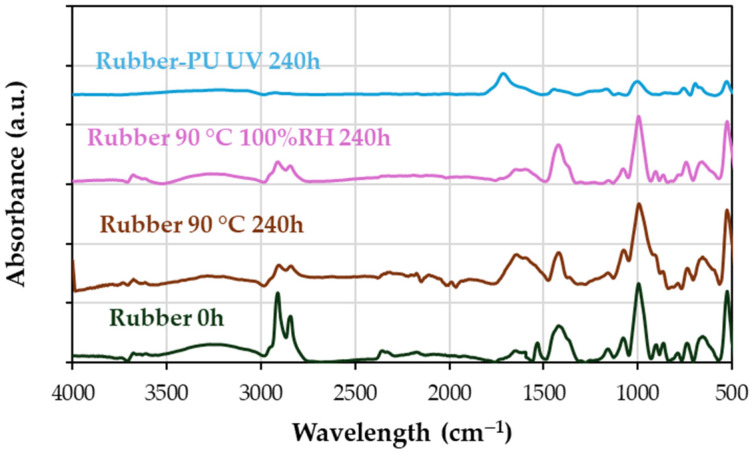
Infrared spectra of the initial rubber (0 h) compared to rubber aged at 90 °C and at 90 °C with 100% RH, both at 240 h, as well as PU-coated rubber at 240 h in a UV chamber.

Comparing the initial rubber with the rubber aged at 90 °C for 240 h (Figure 8b and Figure 11):

- Methyl groups in the range between 2800 and 3000 cm^−1^ disappear.
- The region around 1000 cm^−1^ increases, so too the region between 1500 and 1600 cm^−1^, indicating the formation of double bonds due to free radicals induced by heat. Vinyl and butadiene groups increase due to the formation of double bonds for thermal oxidation in backbone chains [54].
- The stiffening of chains due to crosslinking aligns with the loss of elasticity observed in tensile tests (Table 3, Figure 8b).

In combined aging conditions at 90 °C and 100% RH at 240 h (Figure 11),

- Loss of C-H bonds leads to decreased methylene groups.
- No significant increase in the 1000 cm^−1^ region occurs, maintaining a similarity with the initial rubber.
- A slight increase in hydroxyl groups (3000 to 3500 cm^−1^) correlates with a plasticizing effect, reducing stiffness and slightly preserving elasticity compared to thermal aging alone.

For polyurethane-coated rubber aged under UV light at 240 h (Figure 11),

- Methylene peaks between 2800 and 3000 cm^−1^ disappear.
- A new peak at 1735 cm^−1^, corresponding to urethanes, becomes visible.
- Stiffness increases, as seen in Figure 7, but elasticity decreases due to UV-induced crosslinking.

#### 4.3.3. FTIR Insights into Advanced Aging

**Figure 12:**
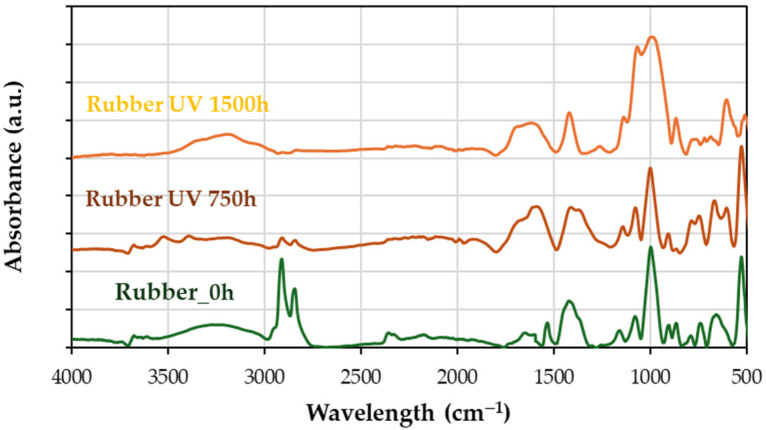
Infrared spectra of the initial rubber (0 h) compared to rubber aged in UV chamber over time 1500 h.

UV aging (Figure 12) reveals the following:

- At 750 h, a significant decrease in methylene groups (2800–3000 cm^−1^) is observed, with stiffness effects seen in Figure 8a.
- At 750 h, between 1500 and 1650 cm^−1^, there is a significant increase, corresponding to double bonds in vinyl and butadiene groups and/or carbonyl groups, with a stiffness effect with a loss of elasticity (Figure 8a)
- At 1500 h, hydroxyl content increases (3000–3500 cm^−1^ and the peak at 1030 cm^−1^), correlating with enhanced plasticity and partially recovered elasticity (Figure 8a).
- The formation of free radicals during UV exposure leads to crosslinking, reducing elasticity, as indicated by a pronounced peak around 1000 cm^−1^.

**Figure 13:**
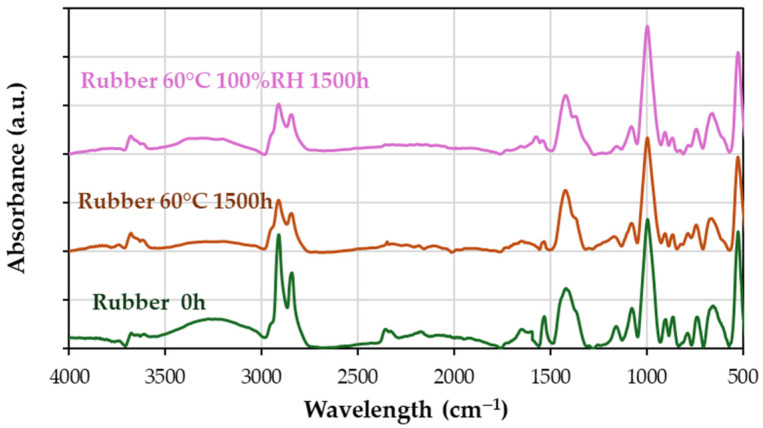
Infrared spectra of the initial rubber (0 h) compared to rubber aged at 60 °C and combined effect of 60 °C and under the combined effect of 60 °C and 100% RH at 1500 h.

FTIR spectra of rubber aged at 60 °C and combined aging at 1500 h (Figure 13) show

- An increase around 900 cm^−1^, a peak associated with double bonds. The peak corresponds to the C=C asymmetric stretching vibrations of double bonds in dienes at 1530 cm^−1^ decreases in the aged samples.
- A decrease in methylene peaks in the region between 2800 and 3000 cm^−1^ and in the peaks at 1030 and 1075 cm^−1^, C-S and C-S-C groups, consistent with the formation of free radicals and subsequent crosslinking reactions. More double bonds result in greater stiffness and a reduction in elasticity, as observed in Table 4 and Figure 8c,d.
- A slight increase in the hydroxyl region (3000 to 3500 cm^−1^), suggesting a minor plasticizing effect that could partially mitigate the loss of elasticity under combined aging conditions.

**Figure 14:**
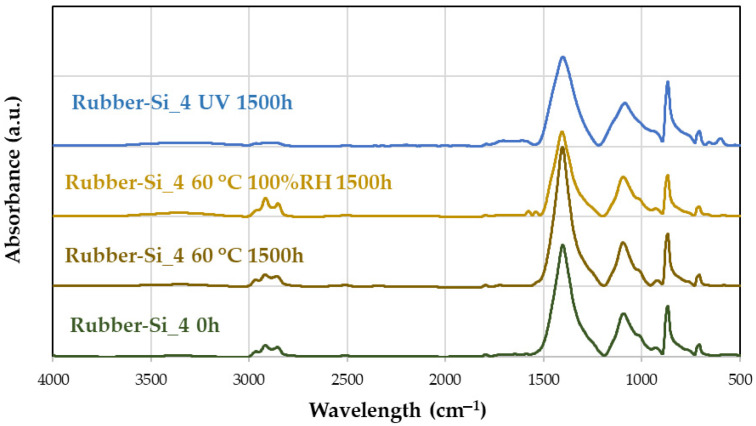
Infrared spectra of the initial Si_4-coated rubber (0 h) compared to Si_4-coated rubber aged at 60 °C, under the combined effect of 60 °C and 100% RH, and UV light at 1500 h.

Silicone-coated rubber (Figure 14) shows five regions of interest:

- Peaks at 700 cm^−1^ and 860 cm^−1^ correspond to Si-(CH_3_)_3_ and Si-(CH_3_)_2_ vibrations, respectively [55].
- The siloxane band (Si-O-Si) around 1060 cm^−1^ [52,55] remains consistent across aging conditions.
- Double bonds from aromatic rings and Si-CH=CH_2_ vibrations overlap at 1390 cm^−1^ [52,55], with aging effects leading to variations in this region.
- Methylene peaks decrease significantly or disappear under UV aging, indicating crosslinking reactions.

In general, all the peaks suggest that only silicone is being analyzed, not rubber. If this is the case, the methylene groups would be bonded to the silicon, equivalent to the CH_3_ groups attached to Si at 700 cm^−1^ and 860 cm^−1^. A similar process may occur in rubber, especially under UV radiation, where the methylene groups decrease or disappear due to crosslinking reactions between double bonds and free radicals. These newly formed double bonds increase stiffness and decrease elasticity (Figure 9b for the aging condition at 60 °C). In the other two conditions (Figure 9a,c), elasticity decreases, but the material exhibits minimal stiffening, following a trend similar to that of uncoated rubber.

It is important to note that UV aging is much more aggressive than aging with temperature and humidity, even though both treatments were applied for the same amount of time. Specifically, 1500 h of UV light is equivalent to 9000 h of midday sunlight exposure, as 20 min in a chamber equals 2 h of sun exposure [56]. Therefore, it is much more aggressive aging.

In light of these findings, the implications of surface treatments and coatings on the durability and environmental sustainability of rubber-based materials warrant further consideration. The proposed approach significantly reduces the environmental impact of rubber waste by limiting its use to the tire casing, complemented by the application of a durable silicone tread. Plasma treatments, such as APPT and silicone coatings, are cost-effective solutions that enhance the durability and performance of the casing, reducing the need for frequent replacements.

These technologies are not novel in the automotive sector, as APPT is widely employed to improve adhesion and surface preparation in plastic components. Thus, their integration into tire manufacturing would require minimal adaptation of existing processes, enabling an efficient and sustainable solution with immediate industrial applicability.

## 5. Conclusions

APPT plasma treatment increases the number of polar groups in polymers, generating hydroxyl and carbonyl groups in rubber. This increases the surface polarity by 95%, improving adhesion and cleaning the surface of contaminants like carbon black. This treatment, which lasts approximately 2 h, results in cohesive failures due to improved adhesion.

Regarding rubber aging, under the three aging conditions studied (at 60 °C, combined effect of 60 °C and 100% RH, and UV light), an increase in stiffness and a reduction in elasticity are observed. Specifically, elasticity is reduced by up to 55%, 59%, and 30% of the initial value, respectively. This is due to the disappearance of the -CH2- groups through free radical formation during thermal oxidation, which promotes crosslinking of the chains, leading to increased stiffness and decreased elasticity, as correlated by FTIR results and tensile tests. However, with UV light, partial recovery of elasticity (17%) is observed at 1500 h, indicating some plasticity, caused by hydroxyl groups.

Silicone, compared to other coatings like polyurethane, shows greater resistance to UV radiation, as well as to factors like temperature and humidity, making it more suitable for applications such as low rolling resistance tires. Silicone coatings reduce stiffness under thermal aging and UV radiation, with no significant color change and lower hardness, unlike polyurethane, which experiences greater loss of elasticity, color changes, and increased hardness. The analysis of the four silicone-based coatings does not show significant variations that justify the choice of one over the others in terms of variations in mechanical behavior. For this reason, the one that has shown the greatest ease of use and application has been chosen: the Si_4 coating.

While the results demonstrate the technical feasibility and advantages of plasma treatment and silicone coatings, certain limitations must be acknowledged. The environmental and economic feasibility of these coatings has not been fully quantified. However, as discussed, plasma treatment and silicone coatings are cost-effective and sustainable solutions compared to smart tires. The scalability of the findings is supported by the widespread use of APPT in automotive manufacturing for adhesion and surface preparation, suggesting that its application in tire casing production would be straightforward and highly adaptable.

Future work will focus on testing the complete smart tire system in full durability trials to evaluate its long-term performance under real-world conditions.

## Figures and Tables

**Table 1:** Characteristics of the coating used in this study.

Named as:	Properties
Si_1	1-component liquid silicone: Colorless, transparent, specially designed for rubber.
PU_1	1-component polyurethane: Colorless, transparent with a glossy finish, good adhesion, and pigment-compatible.
PU_3	PU_1 with 30% catalyst: Colorless.
PU_4	2-component PU: Colorless, high-viscosity, transparent with a matte finish, pleasant to the touch, and pigment-compatible at 20% by weight.
Si_2	1-component black silicone: High-viscosity sealant with excellent performance under environmental conditions.
Si_3	2-component grey silicone: High viscosity, fast curing, with strong adhesion and mechanical performance in adverse environmental conditions.
Si_4	1-componet liquid silicone: Based on MS technology, waterproofing, roof repair, and excellent resistance to UV radiation.

**Table 2:** Percentage composition and elemental ratio of the main components detected on the surface of untreated rubber and rubber after APPT treatment by XPS.

Element	C1s	O1s	N1s	S2p	Si2p	O/C	N/C	S/C	Si/C
NR-SBR	%								
Untreated rubber	84.14	10.84	0.98	0.61	3.43	0.129	0.012	0.007	0.041
APPT treated rubber	67.99	23.85	2.66	1.21	4.29	0.351	0.039	0.018	0.063

**Table 3:** Variation in tensile strength at 240 h for uncoating rubber. The reported value represents the average of the tested samples.

	Time (240 h)	
Temperature (°C)	Tensile Strength (MPa)	Strain at Break (%)
22	2.5 ± 0.2	99 ± 3
40	2.8 ± 0.5	95 ± 4
60	2.7 ± 0.3	64 ± 4
90	2.9 ± 0.3	0.8 ± 0.4

**Table 4:** Variation in the tensile strength of rubber under UV exposure, temperature, and combined temperature and humidity. The reported value represents the average of the tested samples.

	Rubber					
	UV		Temperature (60 °C)		60 °C + RH 100%	
Time (h)	Tensile Strength (MPa)	Strain at Break (%)	Tensile Strength (MPa)	Strain at Break (%)	Tensile Strength (MPa)	Strain at Break (%)
0	2.5 ± 0.2	99 ± 3	2.5 ± 0.2	99 ± 3	2.5 ± 0.2	99 ± 3
240	2.8 ± 0.1	65 ± 5	2.9 ± 0.1	64 ± 5	2.8 ± 0.1	58 ± 4
750	2.9 ± 0.1	47 ± 4	2.4 ± 0.2	40 ± 2	3.4 ± 0.2	39 ± 2
1500	3.0 ± 0.3	70 ± 4	3.1 ± 0.3	45 ± 4	2.2 ± 0.1	41 ± 3

**Table 5:** Influence of silicone coating on rubber aging. The reported value represents the average of the tested samples.

		Coating Effect					
		UV		Temperature (60 °C)		60 °C + RH 100%	
Material	Time (h)	Tensile Strength (MPa)	Strain at Break (%)	Tensile Strength (MPa)	Strain at Break (%)	Tensile Strength (MPa)	Strain at Break (%)
Rubber	0	2.5 ± 0.2	99 ± 3	2.5 ± 0.2	99 ± 3	2.5 ± 0.2	99 ± 3
Rubber	1500	3.0 ± 0.3	70 ± 4	3.1 ± 0.3	45 ± 4	2.2 ± 0.3	41 ± 3
Rubber + Si_4	1000	2.4 ± 0.2	55 ± 5	3.0 ± 0.3	46 ± 4	2.0 ± 0.3	41 ± 6
Rubber + Si_4	1500	2.3 ± 0.3	57 ± 4	2.5 ± 0.4	47 ± 2	2.8 ± 0.5	49 ± 5

**Table 6:** Color variation under UV radiation over time for uncoated rubber and rubber coated with polyurethane and silicone.

Material	Time in UV Chamber (h)	L*	a*	b*	Δ*E*	C*	*h*
Rubber	0	24.04	−0.16	−1.05	0.00	1.06	3.29
	240	26.52	0.58	1.57	3.68	1.67	3.50
	1500	25.29	0.20	0.34	1.90	0.39	3.67
Rubber PU_1	0	24.34	−0.04	−0.54	0.00	0.54	3.14
	240	25.00	0.07	−0.01	0.85	0.07	4.57
	1500	24.55	−0.04	−0.86	0.38	0.86	6.24
Rubber_Si_4	0	27.32	−0.31	−2.61	0.00	2.63	3.26
	240	28.19	−0.25	−2.68	0.87	2.69	3.23
	1500	28.81	−0.28	−2.62	1.49	2.63	3.25

C* = Chroma or saturation or intensity of color; Δ*E* = Color divergence; *h* = Chromaticity angle or real chromaticity; L* = Luminosity (black–white), being L*, a* and b* the coordinates.

**Table 7:** Shore A hardness variation under UV radiation over time for uncoated rubber and rubber coated with polyurethane and silicone.

Time in UV Chamber (h)	Shore Hardness A (SHA)		
	Rubber	PU_1	Si_4
0	75	74	30
240	75	82	38
1500	79	95	10

## Data Availability

The original contributions presented in this study are included in the article. Further inquiries can be directed to the corresponding authors.
